# High Beclin-1 and ARID1A expression corelates with poor survival and high recurrence in intrahepatic cholangiocarcinoma: a histopathological retrospective study

**DOI:** 10.1186/s12885-019-5429-3

**Published:** 2019-03-08

**Authors:** Chao Bi, Mei Liu, Weiqi Rong, Fan Wu, Yang Zhang, Shengtao Lin, Yunhe Liu, Jianxiong Wu, Liming Wang

**Affiliations:** 1 Department of Hepatobiliary Surgery, National Cancer Center/National Clinical Research Center for Cancer /Cancer Hospital, Chinese Academy of Medical Sciences and Peking Union Medical College, 17 Panjiayuan Nanli, Chaoyang District, Beijing, 100021 China; 2 Laboratory of Cell and Molecular Biology and State Key Laboratory of Molecular Oncology, National Cancer Center/National Clinical Research Center for Cancer /Cancer Hospital, Chinese Academy of Medical Sciences and Peking Union Medical College, 17 Panjiayuan Nanli, Chaoyang District, Beijing, 100021 China

**Keywords:** Intrahepatic cholangiocarcinoma, Beclin-1, ARIDIA, IDH1, CA9

## Abstract

**Background:**

Although surgical resection provides a cure for patients with intrahepatic cholangiocarcinoma (ICC), the risk of mortality and recurrence remains high. Several biomarkers are reported to be associated with the prognosis of ICC, including Beclin-1, ARID1A, carbonic anhydrase IX (CA9) and isocitrate dehydrogenase 1 (IDH1), but results are inconsistent. Therefore, a histopathological retrospective study was performed to simultaneously investigate the relationship of these four potential biomarkers with clinicopathological parameters and their prognostic values in patients with ICC.

**Methods:**

A total of 113 patients with ICC were enrolled from Cancer Hospital of Chinese Academy of Medical Sciences between January 1999 and June 2015. The expression of Beclin-1, ARID1A, IDH1 and CA9 were determined by immunohistochemical staining. The prognostic values of the four biomarkers were analyzed by Cox regression and the Kaplan-Meier method.

**Results:**

Beclin-1, ARID1A, CA9 and IDH1 were highly expressed in ICC tumor tissues. Higher mortality was positively associated with Beclin-1 expression (HR = 2.39, 95% CI = 1.09–5.24) and higher recurrence was positively associated with ARID1A expression (HR = 1.71, 95% CI = 1.06–2.78). Neither CA9 nor IDH1 expression was significantly associated with mortality or disease recurrence. Kaplan-Meier survival curves showed that ICC patients with higher Beclin-1 and ARID1A expression had a lower survival rate and a worse recurrence rate than patients with low Beclin-1 and ARID1A expression (*p* < 0.05).

**Conclusions:**

High Beclin-1 and ARIDIA expression are strongly associated with poor prognosis in ICC patients, and thus Beclin-1 and ARID1A should be simultaneously considered as potential prognostic biomarkers for ICC patients.

**Electronic supplementary material:**

The online version of this article (10.1186/s12885-019-5429-3) contains supplementary material, which is available to authorized users.

## Background

Intrahepatic cholangiocellular carcinoma (ICC) is an aggressive cancer that arises from the epithelial bile ducts in the liver and has a very poor prognosis [1]. ICC accounts for 5 to 30% of all primary liver malignancies, and its incidence has been rising over the past few decades [2]. ICC often occurs in cirrhotic and non-cirrhotic livers because of chronic hepatitis, metabolic syndrome and the lack of clear etiological risk factors [3, 4]. Although the reported incidence of ICC is about one to two cases per 100,000 patients in the Western world [5–7], the cancer-related mortality of ICC is increasing, and the 5-year survival rate is less than 20% [8, 9]. Currently, surgical resection is the regular treatment for patients with ICC. However, postoperative resection, conventional chemotherapy and radiotherapy do not significantly improve long-term survival, and there remains a high recurrence rate and metastasis [10–14]. Thus, in past studies, many potential biomarkers for the diagnosis of ICC have been identified. In addition, several studies identified clinicopathological risk factors for ICC recurrence or mortality, such as age, tumor size, number of tumors, lymph node metastasis, vascular invasion, positive margins and pre-existing liver disease [15–17]. However, few studies have systematically and simultaneously investigated the prognostic values of these biomarkers through histopathology.

The disease staging and prognosis of ICC are mainly based on the pathological features of the tumor. Although surgical resection combined with cytotoxic chemotherapy is the standard treatment for ICC, prognosis remains poor, with a median survival of about 1 year [18]. Thus, new and effective molecular targets to identify ICC are urgently needed. Recent studies suggested that tumor cells activate autophagy in response to cellular stress [19–21] shed new light on potential targets for ICC. In addition to inhibiting autophagy by pharmacological agents such as chloroquine [22], it is also important to target potential autophagic biomarkers. In addition to autophagy, several reports which analyzed ICC tissues found significant differences in biomarkers between intrahepatic and extrahepatic cholangiocarcinoma, such as ARID1A, IDH1 and TP53 [23–25].

Autophagy, a major intracellular degradation system, is involved in many pathological and physiological functions of organisms. However, its promoting and suppressing effects in tumorigenesis, inflammatory responses, apoptosis and reactive oxygen species generation make autophagy a double-edged sword in the cancer setting [26–29]. Autophagy has been reported to promote tumor cell survival, tumor progression and metastatic tumor recurrence [30, 31]. The mammalian autophagy gene Beclin-1 plays an important role in the localization of autophagic proteins to a pre-autophagosomal structure, and thus Beclin-1 can coordinately regulate the autophagy activation and endocytic trafficking of autophagosomes [32]. However, recent studies found that Beclin-1 was negatively correlated with disease progression in cholangiocarcinoma patients [33, 34]. Lower Beclin-1 expression was associated with poor overall survival (OS) and progression-free survival (PFS). This association suggests that, in addition to Beclin-1, a variety of biological markers should be considered simultaneously in the identification of cancer prognosis and treatment, particularly ICC.

Recently, several biological markers were also reported to be associated with the prognosis of ICC, such as AT-rich interactive domain 1A (ARID1A), hypoxia-regulated carbonic anhydrase IX (CA9) and isocitrate dehydrogenase 1 (IDH1). ARID1A is generally considered as a tumor-suppressor gene because reduced expression of ARID1A has been found in a variety of tumors and correlated with tumor progression. Lower ARID1A expression was associated with a lower OS rate and lower disease-free survival rate in ICC [35]. CA9 is a known prognostic marker for many cancers, such as ovarian cancer [36, 37], esophageal and gastric adenocarcinomas [38], breast cancer [39] and rectal cancer [40, 41], as well as ICC [42]. CA9 is a transmembrane protein that contributes to microenvironmental acidification and subsequently augments the metastatic potential. Interestingly, CA9 can be released into the circulation by ectodomain shedding, which then influence its biological function [43]. Isocitrate dehydrogenases (IDH) are crucial in cellular metabolism, particularly in tumor cells [44]. IDH1 is responsible for generating NADPH from NADP^+^ by catalyzing the oxidative decarboxylation of isocitrate to 2-ketoglutarate. IDH1 mutation has been identified in a variety of tumor types, such as glioma [45], acute myelogenous leukemia [46, 47], central and periosteal cartilaginous tumors [48] and ICC [49, 50]. IDH1 mutation is associated with a good prognosis in patients with glioma [51, 52] but with a worse prognosis in ICC patients [53].

The study represents the results of a histopathological retrospective study of 113 patients with ICC, and the first simultaneous examination of the expression of Beclin-1, ARID1A, IDH1 and CA9 and their association with ICC. The aim of this study was to elucidate whether Beclin-1, ARID1A, IDH1 and CA9 can be simultaneously used to determine prognosis in ICC.

## Methods

### Patients and tissue specimens

The histopathological retrospective study enrolled 113 patients who received ICC surgery from Cancer Hospital of Chinese Academy of Medical Sciences (CAMS) between January 1999 and June 2015. The study population included patients with complete ICC tumor resection; postoperative pathological confirmation that the tumor was ICC; preoperative image confirmation of no distal metastasis and completed follow-up; and availability of paraffin-embedded tissue samples for subsequent immunohistochemistry analysis. We excluded patients with mixed types of liver cancer, other malignancies, those who died during the perioperative period and those who died of a cause unrelated to cancer. All tumor specimens were collected from Cancer Hospital, CAMS from January 1999 to June 2015. The study was performed in accordance with the Declaration of Helsinki and the protocol and study were approved by the Ethics Committee of CAMS. Informed written consent was obtained from all patients.

### Pre-surgery clinicopathological characteristics

Prior to surgery, the following characteristics were evaluated: gender, age, hepatitis B surface antigen, pre-operative γ-glutamyl transpeptidase, pre-operative total bilirubin, pre-operative alkaline phosphatase, pre-operative tumor markers (CA19–9, CEA and AFP), extent of tumor differentiation, tumor type, number of tumor foci, maximum diameter of the tumor, lymph node metastasis, vascular involvement, intravascular cancer embolus, nerve involvement, involvement of the hepatic capsule, extra-hepatic involvement, tumor necrosis, mucus and hepatic cirrhosis.

### Post-surgery follow-up

Follow-up post-surgery was conducted through hospital and/or telephone visits. Patients were followed until death or October 8, 2015. Patients had chest X-ray, enhanced computed tomography (CT) and analysis of the presence of tumor markers (CA19–9, CEA and AFP) every three months during the first two years following surgery. If abnormalities were observed or recurrence was assessed by imaging and the presence of tumor markers, biopsy or surgery was performed to confirm diagnosis. The treatment strategy for recurrence was mainly based on the comprehensive consideration of the tumor characteristics, liver function and the patient’s general condition. Local curative treatment consisted of re-hepatectomy and radiofrequency ablation (RFA) and systemic palliative treatment, such as molecular targeted therapy and chemotherapy were performed as alternative methods for recurrence treatment. RFS was defined as the time from the date of surgery to the date of detected recurrence or last follow up. OS was defined as the time from the date of surgery to the date of death or last follow up.

### Score of immunohistochemistry staining

Immunohistochemistry was performed on paraffin embedded tissue samples. Briefly, tissues were sectioned into 5 μm thickness. After deparaffinization and hydration, antigen retrieval was performed by incubating with 3% hydrogen peroxide to block endogenous peroxidase. After washing and bovine serum albumin blocking, the specimen was incubated with the primary antibody at 4 °C overnight. Primary antibodies used in this study included the following: for Beclin-1, sc-11,427 H300 (1:100, Santa-Cruz Biotechnology, Santa-Cruz, CA, USA), for ARIDIA, sc-32,761 PSG3 (1:200, Santa-Cruz Biotechnology), for IDH1, Clone OTI24A2 (1:150, Origene Technologies, Rockville, MD, USA) and for CA9, Clone OTI1G7 (1:150, Origene Technologies). Sections were then washed with phosphate-buffered saline, incubated with a PV-9000 Polymer Detection System (PV-9000, GBI Labs, Mukilteo, WA, USA) and color-related with 3,3′-diaminobenzidine solution (ZLI-9018, Zhongshan, Beijing, China). The staining intensity and proportion of positive cells were determined in the same section. Positive cells had light yellow or brown granules. Staining intensity was determined according to the staining characteristics of most cells (contrast to the background) and was scored as follows: no staining, 0 (None); light yellow, 1 (Weak); brown yellow, 2 (Moderate); brown, 3 (Strong). Ten fields were randomly selected at a high magnification (400×), 300 cells were counted in each field and the proportion of positive cells was determined and scored as follows: 0, 0–10% positive cells; 1, 11–25% positive cells; 2, 26–50% positive cells; 3, > 50% positive cells. The staining intensity and proportion of positive cells were determined for each field, and the total score was the sum of both scores: 0, negative (grade 0); 1–2, weakly positive (grade 1); 3–4, moderate positive (grade 2); 5–6; strong positive (grade 3). All sections were scored independently by two experienced pathologists.

### Statistics analysis

All demographic and cancer characteristics were presented as categorical variables, and the differences in Beclin-1, ARID1A, IDH1 and CA9 expression by groups were analyzed by Pearson’s chi-squared test. Death and disease recurrence rates were estimated according to Beclin-1, ARID1A, IDH1 and CA9 expression. Cox’s proportional hazards regression model was constructed to estimate the association of death or disease recurrence with each demographic and cancer characteristics according to Beclin-1, ARID1A, IDH1, and CA9 expression. Demographic and cancer features with significantly higher crude hazard ratios (HRs) for death or recurrence risk were further included in the multiple regression model to explore the adjusted HRs for the different factors. Survival curves for OS and RFS were presented using the Kaplan-Meier approach. Log-rank tests were performed to examine differences in OS and RFS rates by levels of expression of Beclin-1 and ARID1A.

## Results

### Higher Beclin-1 expression is associated with higher mortality and recurrence

**Fig. 1:**
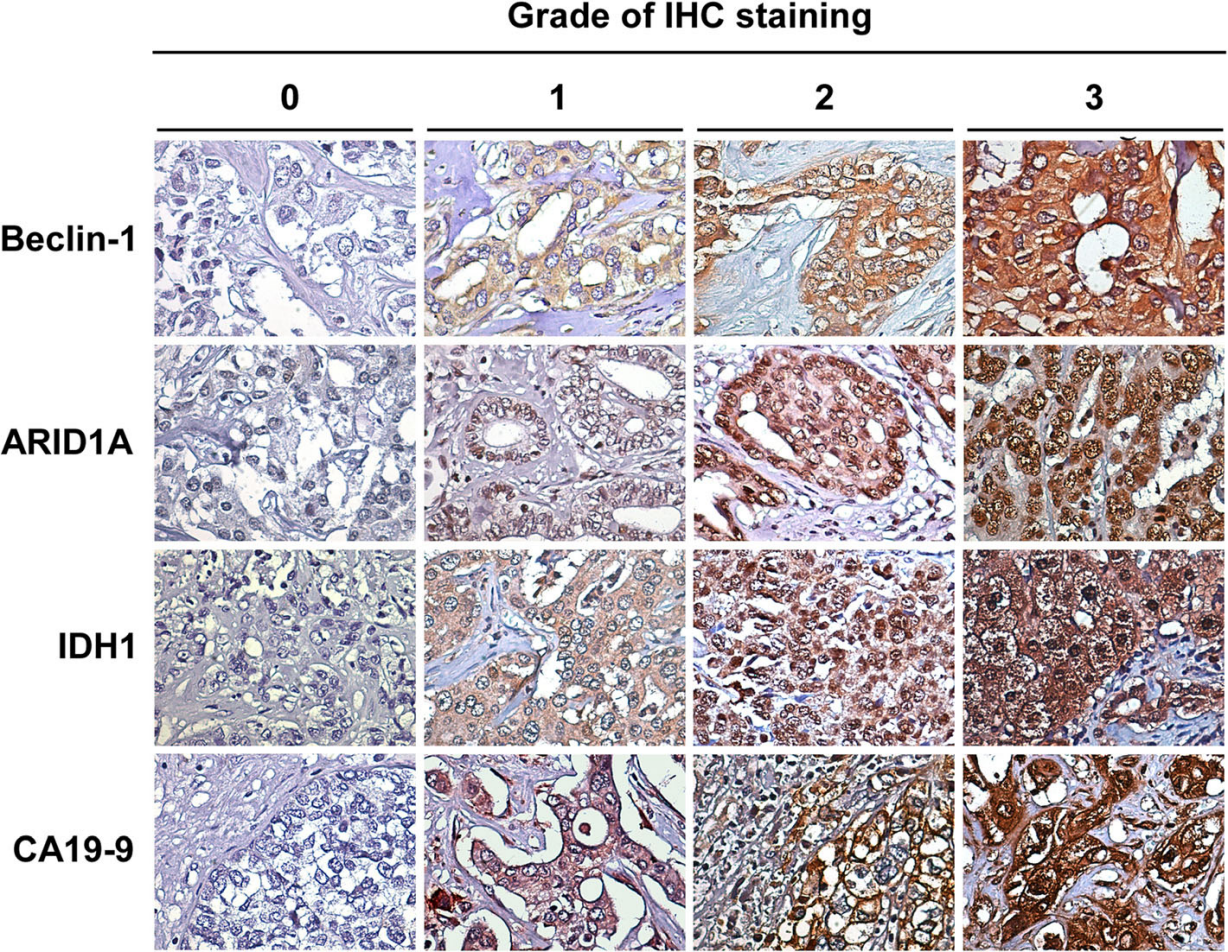
Representative immunohistochemical staining images of Beclin-1, ARIDIA, IDH1 and CA9 protein expression. Grade of expression were classified by IHC staining intensities: grade 0 indicates no staining; grade 1 indicates weak staining; grade 2 and 3 indicate moderate and strong staining, respectively

A total of 113 tissue samples from ICC patients were separately stained with specific antibodies to examine the expression levels of Beclin-1, ARIDIA, CA9 and IDH1. Representative examples of positive and negative immunohistochemical staining of Beclin-1, ARID1A, CA9 and DH1 were shown in Fig. 1. The distribution of staining intensities among the four grades were shown in Table 1. Results of immunohistochemical staining showed that the four biomarkers were highly expressed in ICC tumor tissues. A total of 97 (85.9%) and 77 (68.2%) tumor tissues exhibited positive immunohistochemical staining for Beclin-1 and ARID1A, respectively; 96 (85.0%) and 105 (92.9%) samples stained positive for CA9 and IDH1, respectively.

**Table 1:** Distribution of staining intensities of Beclin-1, ARID1A, CA9 and IDH1 in patients with intrahepatic cholangiocarcinoma (*n* = 113)

Staining intensity	Beclin-1	ARID1A	CA9	IDH1
Grade 0	16	36	17	8
Grade 1	32	40	33	24
Grade 2	42	30	35	49
Grade 3	23	7	28	32

To investigate the relationship between clinical prognosis and the four biomarkers, the above 113 cases of ICC patients were followed up. There was no significant association between ICC mortality and the expression of ARID1A, CA9 or IDH1 (Table 2). However, a higher mortality rate was associated with Beclin-1 expression (HR = 2.39, 95% Confidence Interval [CI]: 1.09–5.24). Compared with grade 0, ICC patients with histologic grade 2 of Beclin-1 expression had a 3.02-fold higher risk of mortality (95% CI: 1.32–6.91); those with histological grade 3 had a 2.95-fold higher risk of mortality (95% CI: 1.22–7.12). We further examined the association of disease recurrence and the four biomarkers (Table 3). There was a 2.75- and 3.19-fold increased risk of recurrence in grade 2 and grade 3 Beclin-1 expression, respectively (grade 2, 95% CI: 1.26–6.00; grade 3, 95% CI: 1.38–7.37). In addition to Beclin-1, ARID1A expression was associated with a 1.71-fold higher risk of recurrence (95% CI: 1.06–2.78). No significant association was found between risk of disease recurrence and the expression of CA9 and IDH1.

**Table 2:** The risk of death in ICC patients according to the expression of Beclin-1, ARID1A, IDH1 and CA9

IHC Intensity	Follow-up (Month)	Number of Death	Rate, n/1000 person-month	Crude HR (95% CI)
Beclin-1				
0	688.41	7	10.17	1.0
1	1036.01	18	17.37	1.60 (0.67–3.83)
2	811.01	32	39.46	**3.02 (1.32–6.91)**
3	529.00	18	34.03	**2.95 (1.22–7.12)**
Negative	688.41	7	10.17	1.0
Positive	2376.02	68	28.62	**2.39 (1.09–5.24)**
ARID1A				
0	1225.53	20	16.32	1.0
1	984.01	32	32.52	1.71 (0.98–3.00)
2	712.29	19	26.67	1.49 (0.79–2.80)
3	142.60	4	28.05	1.29 (0.44–3.78)
Negative	1225.53	20	16.32	1.0
Positive	1838.90	55	29.91	1.59 (0.95–2.66)
IDH1				
0	287.39	5	17.40	1.0
1	627.57	17	27.09	1.10 (0.40–2.99)
2	1423.95	32	22.47	1.001 (0.39–2.58)
3	725.52	21	28.94	1.09 (0.41–2.92)
Negative	287.39	5	17.40	1.0
Positive	2777.04	70	25.21	1.05 (0.42–2.61)
CA9				
0	654.60	10	15.28	1.0
1	641.92	24	37.39	1.53 (0.73–3.22)
2	985.60	25	25.37	1.08 (0.52–2.25)
3	782.31	16	20.45	0.96 (0.44–2.12)
Negative	654.60	10	15.28	1.0
Positive	2409.83	65	26.97	1.17 (0.60–2.27)

Bold indicates statistical significance, p < 0.05

**Table 3:** The risk of recurrence in ICC patients according to the expression of Beclin-1, ARID1A, IDH1 and CA9

IHC intensity	Follow-up (Month)	Number of Recurrence	Rate, n/1000 person-month	Crude HR (95% CI)
Beclin-1				
0	581.14	8	13.77	1.0
1	812.20	22	27.09	1.55 (0.69–3.50)
2	496.95	35	70.43	**2.75 (1.26–6.00)**
3	310.27	20	64.46	**3.19 (1.38–7.37)**
Negative	581.14	8	13.77	1.0
Positive	1619.42	77	47.55	**2.27 (1.09–4.75)**
ARID1A				
0	994.59	23	23.13	1.0
1	641.70	34	52.98	**1.73 (1.01–2.94)**
2	480.94	23	47.82	1.76 (0.98–3.14)
3	83.33	5	60.00	1.47 (0.56–3.89)
Negative	994.59	23	23.13	1.0
Positive	1205.97	62	51.41	**1.71 (1.06–2.78)**
IDH1				
0	258.99	5	19.31	1.0
1	418.98	20	47.74	1.56 (0.58–4.16)
2	1071.72	35	32.66	1.14 (0.44–2.90)
3	450.87	25	55.45	1.38 (0.53–3.63)
Negative	258.99	5	19.31	1.0
Positive	1941.57	80	41.20	1.29 (0.52–3.20)
CA9				
0	541.80	12	22.15	1.0
1	420.68	26	61.80	1.12 (0.56–2.23)
2	695.73	27	38.81	0.88 (0.44–1.75)
3	542.35	20	36.88	0.91 (0.44–1.86)
Negative	541.80	12	22.15	1.0
Positive	1658.76	73	44.01	0.96 (0.52–1.77)

Bold indicates statistical significance, p < 0.05

### Association of clinical features with the expression of Beclin-1 and ARID1A

The patients’ demographic characteristics and the expression of Beclin-1 and ARID1A were listed in Additional file 1: Table S1. The distribution of most baseline characteristics was similar between the Beclin-1 negative and Beclin-1 positive group and between the ARID1A negative and ARID1A positive group. The clinical variables considered as potential predictors of risk of death and recurrence in ICC patients were listed in Additional file 1: Table S2. A multivariate analysis was performed to analyze the independent association of Beclin-1 and ARID1A with the risk of death or disease recurrence in ICC patients. As shown in Table 4, Beclin-1 expression was strongly associated with the risk of death (adjusted HR = 2.51, 95% CI = 1.05–5.99), while ARID1A was significantly correlated with the risk of death (adjusted HR = 1.95, 95% CI = 1.09–3.47) and the risk of disease recurrence (adjusted HR = 2.08, 95% CI = 1.23–3.51).

**Table 4:** Univariate and multivariate Cox regression analyses Beclin-1 and ARID1A for the risk of death and recurrence in ICC patients

		Death		Recurrence	
		Crude HR	adjusted HR ^a^	Crude HR	adjusted HR ^b^
Beclin-1	Negative	1.0	1.0	1.0	1.0
	Positive	**2.39 (1.09–5.24)**	**2.51 (1.05–5.99)**	**2.27 (1.09–4.75)**	1.85 (0.85–4.06)
ARID1A	Negative	1.0	1.0	1.0	1.0
	Positive	1.59 (0.95–2.66)	**1.95 (1.09–3.47)**	**1.71 (1.06–2.78)**	**2.08 (1.23–3.51)**

Bold indicates statistical significance, p < 0.05

^a^model controlled for age, GGT before surgery, ALP before surgery, CEA before surgery, number of lesions, tumor size, lymphatic metastasis, nerve invasion, involving extrahepatic tissue, mucus, tumor generalization, TNM stage.

^b^ model controlled for GGT before surgery, ALP before surgery, CEA before surgery, number of lesions, tumor size, lymphatic metastasis, involving extrahepatic tissue, necrosis, mucus, TNM stage.

### Association of Beclin-1 and ARID1A expression with prognosis

**Fig. 2:**
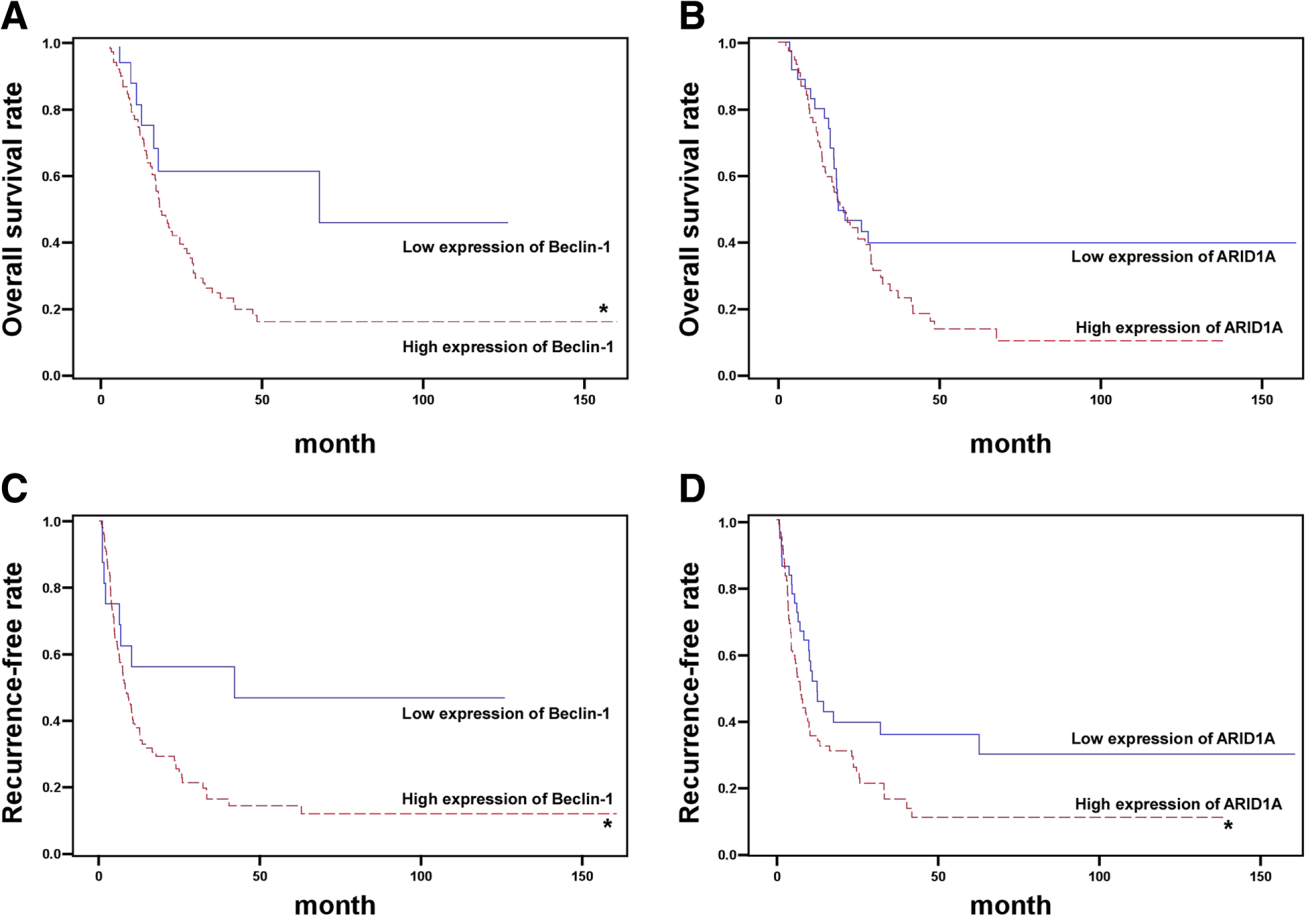
Kaplan-Meier curves for time to survival and recurrence-free rate in patients with ICC. Overall survival of ICC patients according to the level of Beclin-1 (**a**) and ARID1A (**b**). Recurrence-free rate of ICC patients according to the level of Beclin-1 (**c**) and ARID1A (**d**)

**Fig. 3:**
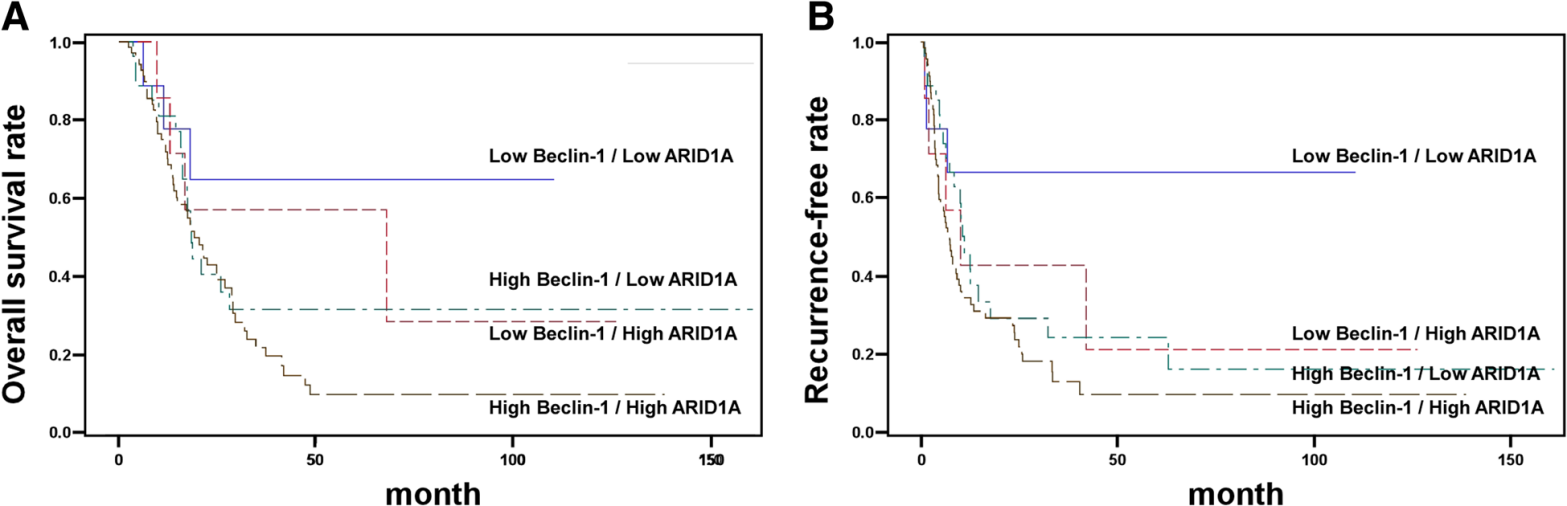
Relationship between patient survival and recurrence and the expression of Beclin-1 and ARID1A. Overall survival rate (**a**) and recurrence-free rate (**b**) in ICC patients with high or low Beclin-1 and ARID1A

Kaplan-Meier survival curves showed that high levels of Beclin-1 were associated with poor prognosis. The OS rate for those with high levels of Beclin-1 expression was significantly shorter than that for those with low levels of Beclin-1 expression (*p* < 0.05; Fig. 2a). In contrast, the OS rate for those with high levels of ARID1A expression was lower than that of those with low ARID1A expression, but the difference was not statistically significant (*p* = 0.07; Fig. 2b). On the other hand, high levels of expression of both Beclin-1 and ARID1A were significantly associated with lower RFS rates when compared with low Beclin-1 and ARID1A expression (p < 0.05; Fig. 2c and d). Taking all cases together, we found that ICC patients with high levels of expression of both Beclin-1 and ARID1A had the lowest OS rate (Fig. 3a) and the highest disease recurrence rate (Fig. 3b). The expression of CA9 and IDH1 in these double positive Beclin-1 and ARID1A patients was further analyzed. Interestingly, we found that IDH1 and CA9 were both positively expressed in most patients with positive Beclin-1 and ARID1A. Of the 70 patients with double positive expression of Beclin-1 and ARID1A, 59 showed positive expression of IDH1 and CA9. However, the difference with other groups was not statistically significant (*p* = 0.732 for mortality and *p* = 0.505 for recurrence). Collectively, the study indicated that Beclin-1 and ARID1A, but not CA9 or IDH1, can be used simultaneously as prognostic factors for clinical outcomes in ICC patients.

## Discussion

ICC is a malignant neoplasm in the biliary duct system with a high mortality rate. Despite continuous improvement in surgical techniques, the prognosis for ICC remains poor. Even after combined surgical resection, radiotherapy and adjuvant chemotherapy, the recurrence rate is still high because of regional invasiveness and distant metastasis. Although several valuable biomarkers have been reported to be associated with the prognosis of ICC, few studies have systematically explored the prognostic value of these biomarkers. These potential biomarkers include serum carbohydrate antigen 19–9 (CA19–9), interleukin-6 (IL-6), Beclin-1, ARID1A, CA9 and IDH1. However, we found that levels of CA19–9 and IL-6 were easily influenced by factors other than the presence of ICC. The serum level of CA19–9 was also increased in patients with other tumors such as gastric cancer or pancreatic cancer. Although the inflammatory cytokine IL-6 was increased in ICC patients, it is easily elevated in response to other inflammatory stimuli. Thus, this study excluded CA19–9 and IL-6, and conducted a histopathological retrospective study to assess simultaneously the prognostic value of Beclin-1, ARIDIA, IDHA and CA9 in the OS and disease recurrence of patients with ICC.

Our study found that ICC patients with higher Beclin-1 expression had a greater risk of mortality and disease recurrence. Interestingly, Beclin-1 is an essential protein for autophagy which play multiple roles in tumor progression and suppression. Existing reports on the prognostic role of autophagy or autophagy-associated proteins such as Beclin-1 in tumor progression are often contradictory. Higher Beclin-1 expression was suggested to be a positive prognostic factor for patients with colorectal cancers [54], oral tongue squamous cell carcinoma [55] and natural killer T-cell lymphoma [56]. However, other reports found higher expression of Beclin-1 associated with a poor prognosis in endometrial adenocarcinomas [57] or nasopharyngeal carcinoma [58]. As to the association of Beclin-1 and cholangiocarcinoma, our histopathological retrospective study found that higher expression of Beclin-1 was significantly associated with a poor OS rate and recurrence-free rate. However, the result is different from that of Dong et al., who reported that lower Beclin-1 expression was associated with worse OS [34]. Although our study included more patients, this difference in results may arise from differences in the included patient characteristics, therapeutic surgery used or the influence of other prognostic factors. Thus, in addition to Beclin-1, other potential prognostic markers such as ARID1A should be included simultaneously in the histopathological determination of OS or RFS.

ARID1A, a member of the switching defective/sucrose non-fermenting complexes, is generally considered a tumor suppressor gene in breast cancer [59], hepatocellular carcinoma [60] and pancreatic cancer [61]. However, recent studies demonstrated that ARID1A has dual roles in both oncogenicity and tumor suppression [62, 63]. ARID1A can promote tumor initiation through CYP450-mediated oxidative damage. Consistent with our study, we found that increased expression of ARID1A was associated with a higher risk of mortality and disease recurrence in ICC patients. However, Yang et al. reported that low expression of ARIDIA correlated with poor prognosis in patients with ICC. The difference may be due to their small number of patients, only 57 ICC patients. In addition, the dual roles of ARID1A may contribute to the difference in results between studies. Therefore, using multiple prognostic factors, especially for contradictory prognostic factors, may avoid inconsistencies.

Recently, CA9 and IDH1 were reported to be diagnostic factors for cholangiocarcinoma [42, 49, 50]. However, our study showed no significant relationship between CA9 or IDH1 and risk of mortality or disease recurrence in ICC patients. Although the expression of IDH1 was largely increased in ICC patients (8 negative vs. 105 positive), IDH1 level was not associated with mortality (HR = 1.05; 95% CI: 0.42–2.61) or disease recurrence (HR = 1.29; 95% CI: 0.52–3.2). It is likely that IDH1 gene mutation, but not the protein level, is the crucial factor contributing to ICC pathogenesis, mortality and disease recurrence. Mutation in the IDH1 gene might not significant alter the protein level; thus, histological staining for IDH1 could not be used to diagnose ICC. On the other hand, the hypoxia-regulated CA9 is not only expressed on the cell surface, but also released into the circulation by ectodomain shedding and functions as an indicator of therapeutic outcome. Of interest, the results of Hektoen et al. demonstrated that an increase in circulating CA9 was associated with a better PFS rate in patients with rectal cancer. In contrast to Korkeila’s immunohistochemical analysis of CA9 [40], disease-free survival was significantly shorter in patients with moderate/strong CA9 staining intensity. This discrepancy may indicate that intracellular and circulating CA9 may contribute to different prognostic outcomes. Thus, it is possible that the level of circulating CA9 may not indicate the prognostic value of immunohistochemical CA9 in ICC patients. This issue warrants further investigation.

Since the results of this study, especially for Beclin-1, contradict those of other studies [33, 34], we should point out that the recognition epitopes of the antibodies used may have led to the observed contradictory findings. However, the Beclin-1 antibody in this study was the same as in the study by Wang et al. (Santa Cruz, sc-11,427 H300). Although Dong et al. used a different Beclin-1 antibody, the antibody was generated by using the same immunogenic region of Beclin-1 (aa 1–300). Thus, the observed contradictory findings of Beclin-1 were likely not caused by the recognition epitope of the Beclin-1 antibody. Of interest, the antibodies used by Korkeila et al. and Gu et al. were generated by aa 359–459 (Abcam, ab15086) and aa 200–300 (Abcam, ab108351) of CA9, respectively [40, 42]. However, the CA9 antibody used in this study (Origene, OTI1G7) was generated from a full-length CA9 immunogen. Structurally, CA9 consists of a transmembrane segment, an N-terminal proteoglycan-like domain, a catalytic domain and an intracytoplasmic portion [64]. Of interest, the immunogenic region used in these studies was the extracellular region of CA9. Whether the recognized epitopes other than the transmembrane segment of CA9 are related to the observed contradictory findings warrants further investigation.

## Conclusion

In summary, to determine the biomarkers associated with mortality and disease recurrence in ICC, the study simultaneously evaluated four controversial prognostic factors: Beclin-1, ARID1A, CA9 and IDH1. Although CA9 and IDH1 did not show great prognostic effects in the risk of mortality and disease recurrence of ICC, both Beclin-1 and ARID1A were highly correlated with poor prognosis in ICC patients. Of importance, patients with high expression of both Beclin-1 and ARID1A had very poor OS and RFS rates. Collectively, the result of this study showed that Beclin-1 and ARIDIA can be simultaneously used as prognostic indicators to predict disease recurrence and mortality in ICC patients.

## Additional file

Additional file 1: **Table S1.** Demographic and clinical characteristics of ICC patients with the expression of Becin-1 and ARID1A (*n* = 113).**Table S2.** Death and recurrence risk with baseline characteristics for patients with intrahepatic cholangiocarcinoma (*n* = 113). (DOCX 32 kb)

## Abbreviations

ARID1A: AT-rich interactive domain 1A
CA9: carbonic anhydrase IX
CAMS: Cancer Hospital of Chinese Academy of Medical Sciences
CT: Computed tomography
ICC: Intrahepatic cholangiocarcinoma
IDH1: Isocitrate dehydrogenase 1
OS: Overall survival
PFS: Progression-free survival
RFA: Radiofrequency ablation
