# Supplementary material for: High Beclin-1 and ARID1A expression corelates with poor survival and high recurrence in intrahepatic cholangiocarcinoma: a histopathological retrospective study
Source: BMC Cancer. 2019 Mar 8;19:213. doi: 10.1186/s12885-019-5429-3 (PMC6408801; doi:10.1186/s12885-019-5429-3)
Supplement: Supplementary file 1 — Table S1. Demographic and clinical characteristics of ICC patients with the expression of Becin-1 and ARID1A (n = 113).Table S2. Death and recurrence risk with baseline characteristics for patients with intrahepatic cholangiocarcinoma (n = 113). (DOCX 32 kb) [file 12885_2019_5429_MOESM1_ESM.docx]

**Additional Files**

**Table S1. Demographic and clicnial characteristics of ICC patients with the expression of Beclin-1 and ARID1A (n=113)**

|  | **Total** | **Beclin-1** | |  | **ARID1A** | | |  | |
| --- | --- | --- | --- | --- | --- | --- | --- | --- | --- |
|  |  | **Negative** | **Positive** | **p-value** | **Negative** | **Positive** | **p-value** | |  |
|  | **N=113** | **N=16** | **N=97** |  | **N=36** | **N=77** |  | |  |
|  |  | **n (%)** | **n (%)** |  | **n (%)** | **n (%)** |  | |  |
| Male | 60 (53.1) | 8 (50.0) | 52 (53.6) | 0.79 | 21 (58.3) | 39 (50.6) | 0.45 | |  |
| Age, year |  |  |  | 0.66 |  |  | 0.99 | |  |
| 0-44 | 10 (8.9) | 1 (6.2) | 9 (9.3) |  | 3 (8.3) | 7 (9.1) |  | |  |
| 45-59 | 59 (52.2) | 10 (62.5) | 49 (50.2) |  | 19 (52.8) | 40 (51.9) |  | |  |
| 60+ | 44 (38.9) | 5 (31.3) | 39 (40.2) |  | 14 (38.9) | 30 (39) |  | |  |
| HBsAg positive | 23 (20.4) | 2 (12.5) | 21 (21.7) | 0.40 | 6 (16.7) | 17 (22.1) | 0.51 | |  |
| GGT before surgery |  |  |  | 0.33 |  |  | 0.99 | |  |
| normal | 44 (38.9) | 8 (50.0) | 31 (37.1) |  | 14 (38.9) | 30 (39.0) |  | |  |
| increase | 69 (61.1) | 8 (50.0) | 61 (62.9) |  | 22 (61.1) | 47 (61.0) |  | |  |
| TBIL before surgery |  |  |  | 0.10 |  |  | 0.14 | |  |
| normal | 104 (92.0) | 13 (81.3) | 91 (93.8) |  | 31 (86.1) | 73 (94.8) |  | |  |
| increase | 9 (8.0) | 3 (18.8) | 6 (6.2) |  | 5 (13.9) | 4 (5.2) |  | |  |
| ALP before surgery |  |  |  | 0.69 |  |  | 0.50 | |  |
| normal | 102 (90.3) | 14 (87.5) | 88 (90.7) |  | 34 (94.4) | 68 (88.3) |  | |  |
| increase | 11 (9.7) | 2 (12.5) | 9 (9.3) |  | 2 (5.6) | 9 (11.7) |  | |  |
| CA19-9 before surgery |  |  |  | 0.01 |  |  | 0.18 | |  |
| normal | 26 (23.0) | 2 (12.5) | 24 (24.7) |  | 5 (13.9) | 21 (27.3) |  | |  |
| increase | 25 (22.1) | 0 (0.0) | 25 (25.8) |  | 7 (19.4) | 18 (23.4) |  | |  |
| unknown | 62 (54.9) | 14 (87.5) | 48 (49.5) |  | 24 (66.7) | 38 (49.4) |  | |  |
| CEA before surgery |  |  |  | 0.29 |  |  | 0.34 | |  |
| normal | 79 (69.9) | 13 (81.3) | 66 (68.0) |  | 23 (63.9) | 56 (72.7) |  | |  |
| increase | 34 (30.1) | 3 (18.8) | 31 (32.0) |  | 13 (36.1) | 21 (27.3) |  | |  |
| AFP before surgery |  |  |  | 0.37 |  |  | 0.77 | |  |
| normal | 98 (86.7) | 15 (93.8) | 83 (85.6) |  | 32 (88.9) | 66 (85.7) |  | |  |
| increase | 15 (13.3) | 1 (6.3) | 14 (14.4) |  | 4 (11.1) | 11 (14.3) |  | |  |
| Cancer differentiation level |  |  |  | 0.38 |  |  | 0.56 | |  |
| poor/ low/ low-median | 52 (46.0) | 9 (56.3) | 43 (44.3) |  | 18 (50.0) | 34 (44.2) |  | |  |
| median/ high | 61 (54.0) | 7 (43.8) | 54 (55.7) |  | 18 (50.0) | 43 (55.8) |  | |  |
| Number of lesions |  |  |  | 0.89 |  |  | 0.75 | |  |
| single | 100 (88.5) | 14 (87.5) | 86 (88.7) |  | 31 (86.1) | 69 (89.6) |  | |  |
| multiple | 13 (11.5) | 2 (12.5) | 11 (11.3) |  | 5 (13.9) | 8 (10.4) |  | |  |
| Tumor size |  |  |  | 0.62 |  |  | 0.98 | |  |
| 5cm>d | 50 (44.2) | 8 (50.0) | 42 (43.3) |  | 16 (44.4) | 34 (44.2) |  | |  |
| d>=5cm | 63 (55.8) | 8 (50.0) | 55 (56.7) |  | 20 (55.6) | 43 (55.8) |  | |  |
| Lymphatic metastasis | 27 (23.9) | 2 (12.5) | 25 (25.7) | 0.25 | 9 (25.0) | 18 (23.4) | 0.85 | |  |
| Peripheral vessel invasion | 15 (13.3) | 1 (6.3) | 14 (14.3) | 0.37 | 4 (11.1) | 11 (14.3) | 0.77 | |  |
| Vessel invasion | 17 (15.0) | 2 (12.5) | 15 (15.5) | 0.76 | 3 (8.3) | 14 (18.2) | 0.17 | |  |
| Nerve invasion | 18 (15.9) | 3 (18.8) | 15 (15.5) | 0.74 | 3 (8.3) | 15 (19.5) | 0.13 | |  |
| Involving the liver capsule | 90 (79.7) | 12 (75.0) | 78 (80.4) | 0.62 | 28 (77.8) | 62 (80.5) | 0.74 | |  |
| Involving extrahepatic tissue | 29 (25.7) | 4 (25.0) | 25 (25.8) | 0.95 | 10 (27.8) | 19 (24.7) | 0.73 | |  |
| Necrosis | 30 (26.6) | 2 (12.5) | 28 (28.9) | 0.17 | 8 (22.2) | 22 (28.6) | 0.48 | |  |
| Mucus | 8 (7.1) | 1 (6.3) | 7 (7.2) | 0.89 | 1 (2.8) | 7 (9.1) | 0.43 | |  |
| Liver cirrhosis | 14 (12.4) | 2 (12.5) | 12 (12.4) | 0.99 | 4 (11.1) | 10 (13.0) | 1.00 | |  |
| Tumor generalization |  |  |  | 0.48 |  |  | 1.00 | |  |
| Lump type | 110 (97.4) | 16 (100) | 94 (96.9) |  | 35 (97.2) | 75 (97.4) |  | |  |
| Infiltration type | 3 (2.6) | 0 (0.0) | 3 (3.1) |  | 1 (2.8) | 2 (2.6) |  | |  |
| TNM stage |  |  |  | 0.57 |  |  | 0.54 | |  |
| I | 54 (47.8) | 10 (62.5) | 44 (45.4) |  | 16 (44.4) | 38 (49.4) |  | |  |
| II | 14 (12.4) | 2 (12.5) | 12 (12.4) |  | 3 (8.3) | 11 (14.3) |  | |  |
| III | 18 (15.9) | 2 (12.5) | 16 (16.5) |  | 8 (22.2) | 10 (13.0) |  | |  |
| IV | 27 (23.9) | 2 (12.5) | 25 (25.8) |  | 9 (25.0) | 18 (23.4) |  | |  |

**Table S2. Death and recurrence risk with baseline characteristics for patients with intrahepatic cholangiocarcinoma (n=113).**

|  | **Death** | **Recurrence** |
| --- | --- | --- |
|  | **Crude HR (95% CI)** | **Crude HR (95% CI)** |
| Gender (Male vs. Female) | 1.15 (0.73-1.82) | 1.25 (0.82-1.93) |
| Age, year (vs. 0-44) |  |  |
| 45-59 | 1.62 (0.64-4.12) | 1.53 (0.65-3.58) |
| 60+ | 2.21 (0.86-5.71) | 1.64 (0.69-3.94) |
| HBsAg (positive vs. negative) | 0.62 (0.34-1.12) | 0.70 (0.41-1.22) |
| **GGT before surgery (Increase vs. normal)** | **2.08 (1.26-3.43)** | **1.69 (1.07-2.67)** |
| TBIL before surgery (Increase vs. normal) | 0.62 (0.27-1.42) | 0.67 (0.31-1.44) |
| **ALP before surgery (Increase vs. normal)** | **3.69 (1.78-7.63)** | **2.61 (1.33-5.13)** |
| CA19-9 before surgery (Increase vs. normal) | 1.49 (0.71-3.14) | 1.01 (0.52-1.94) |
| **CEA before surgery (Increase vs. normal)** | **3.19 (1.95-5.22)** | **2.58 (1.61-4.13)** |
| AFP before surgery (Increase vs. normal) | 0.95 (0.50-1.80) | 0.93 (0.50-1.76) |
| Cancer differentiation level |  |  |
| poor/ low/ low-median | 1.0 | 1.0 |
| median/ high | 0.74 (0.47-1.16) | 0.71 (0.46-1.08) |
| **Number of lesions (multiple vs. single)** | **2.09 (1.09-3.98)** | **2.67 (1.44-4.94)** |
| **Tumor size (d>=5cm vs. 5cm>d)** | **1.69 (1.06-2.69)** | **1.81 (1.17-2.81)** |
| **Lymphatic metastasis (yes vs. no)** | **3.46 (2.07-5.80)** | **2.24 (1.37-3.67)** |
| Peripheral vessel invasion (yes vs. no) | 1.39 (0.75-2.59) | 1.51 (0.83-2.75) |
| Vessel invasion (yes vs. no) | 1.29 (0.66-2.53) | 1.41 (0.78-2.57) |
| **Nerve invasion (yes vs. no)** | **2.18 (1.20-3.95)** | 1.73 (0.98-3.05) |
| Involving the liver capsule (yes vs. no) | 1.46 (0.83-2.57) | 1.60 (0.94-2.72) |
| **Involving extrahepatic tissue (yes vs. no)** | **2.25 (1.38-3.67)** | **1.63 (1.01-2.63)** |
| **necrosis (yes vs. no)** | 1.43 (0.85-2.39) | **2.04 (1.27-3.25)** |
| **mucus (yes vs. no)** | **2.89 (1.37-6.07)** | **2.15 (1.03-4.47)** |
| liver cirrhosis (yes vs. no) | 0.67 (0.31-1.46) | 0.75 (0.38-1.50) |
| **Tumor generalization (infiltration vs. lump)** | **3.64 (1.12-11.82)** | 2.24 (0.70-7.18) |
| TNM stage (vs. I) |  |  |
| **II** | 1.51 (0.71-3.21) | **1.98 (1.00-3.92)** |
| **III** | **2.45 (1.30-4.61)** | **1.88 (1.02-3.46)** |
| **IV** | **4.58 (2.56-8.18)** | **2.88 (1.67-4.97)** |

Bold indicates statistical significance, p<0.05
